# Risk factors associated with acute kidney injury in a pediatric intensive care unit in Addis Ababa Ethiopia: case-control study

**DOI:** 10.1186/s12882-023-03322-y

**Published:** 2023-09-21

**Authors:** Mulualem Keneni, Rajalakshmi Murugan, Ketema Bizuwork, Tesfaye Asfaw, Sosina Tekle, Gadissa Tolosa, Assefa Desalew

**Affiliations:** 1 School of Nursing and Midwifery, College of Health and Medical Sciences, Haramaya University, Harar, Ethiopia; 2 School of Nursing and Midwifery, College of Health Science, Addis Ababa University, Addis Ababa, Ethiopia

**Keywords:** Acute kidney Injury, PICU, Children, Risk factors, Ethiopia

## Abstract

**Background:**

Acute kidney injury (AKI) is a serious health problem in critically ill children. It is associated with poor treatment outcomes and high morbidity and mortality rates. Globally, one in three critically ill children suffers from acute kidney injury. However, limited data are available in Africa, particularly Ethiopia, which highlighting the risk factors related to acute kidney injury. Therefore, this study aimed to identify the risk factors associated with acute kidney injury among critically ill children admitted to the pediatric intensive care unit (PICU) at Tikur Anbessa Specialized Hospital, Addis Ababa, Ethiopia.

**Methods:**

A facility-based unmatched case-control study was carried out on 253 (85 cases and 168 controls) critically ill children admitted to the pediatric intensive care unit from January 2011 to December 2021. Participants were selected using a systematic random sampling technique for the control group and all cases consecutively. Data were collected using a structured checklist. Data were entered using Epi data version 4.6 and analyzed using SPSS version 25. Multivariable analysis was carried out using the adjusted odds ratio (aOR) with a 95% confidence interval (CI) to identify associated factors with acute kidney injury. Statistical significance was set at P < 0.05.

**Results:**

The median age of the participants was two years. Approximately 55.6% of cases and 53.1% of controls were females. The diagnosis of hypertension (aOR = 5.36; 95% CI: 2.06–13.93), shock (aOR = 3.88, 95% CI: 1.85–8.12), exposure to nephrotoxic drugs (aOR = 4.09; 95% CI: 1. 45- 11.59), sepsis or infection aOR = 3.36; 95% CI: 1.42–7.99), nephritic syndrome (aOR = 2.97; 95% CI:1.19, 7.43), and use of mechanical ventilation aOR = 2.25, 95% CI: 1.12, 4.51) were significantly associated factors with acute kidney injury.

**Conclusion:**

The diagnosis of sepsis or infection, hypertension, shock, nephrotoxic drugs, demand for mechanical ventilation support, and nephritic syndrome increased the risk of AKI among critically ill children. Multiple risk factors for AKI are associated with illness and severity. All measures that ensure adequate renal perfusion must be taken in critically ill children with identified risk factors to prevent the development of AKI.

## Introduction

Acute kidney injury (AKI) refers to any sudden decline in kidney function that can be reversible with timely detection and interventions. It is a common complication in children admitted to (PICU) [1–4]. Kidney disease and improving global outcomes (KDIGO) have been established to provide a standardized definition for AKI in children by harmonizing the risk, injury, failure, loss, end-stage (RIFLE), and acute kidney injury network (AKIN) criteria [1, 5]. Staging is based on changes in serum creatinine from baseline or urine output as stages I, II, III, and severe AKI [6]. AKI is one of the most serious global public health problems in the pediatric population [6, 7]. It is highly associated with mortality risk in children worldwide. In children, one-third of the AKI survivors carry the risk of future progression to chronic kidney diseases such as proteinuria, hypertension, and reduced glomerular filtration rate (GFR) may persist in up to 60% of the survivors [1, 3, 8–12].

The International Society of Nephrology revealed that 13.3 million patients develop AKI per year worldwide, and of these, 85% live in low- and middle-income countries (LMICs) [3, 13]. Moreover, KDIGO reported, one in three children experienced AKI during hospital admission, with 30% of incidence among critically ill children [14]. The Assessment of Worldwide AKI Renal Angina and Epidemiology (AWARE) study indicated that 15% of children had AKI on the first day of admission to the PICU, 26.9% had developed AKI within the first seven days of admission, and 11.6% had severe AKI [5, 10, 15, 16].

The risk factors for AKI are not well investigated especially in LMICs [13]. The possible causes of AKI are multidimensional and widely vary from individual to individual and geographical location [1]. The burden of AKI is associated with poor awareness of the public about the possible causes of kidney disease. Some studies indicated that prolonged hospital stay, need for mechanical ventilation, infection, and volume depletion are factors associated with AKI [1, 13]. Children often present to the hospital late, which suggests more severe AKI at admission and an increased risk of death in LMICs [14]. The disease imposes a severe burden of morbidity and mortality, with a major economic effect on healthcare expenditure worldwide, especially in low-resource settings [4, 14, 17–20]. Studies by the International Society of Nephrology imply that wide gaps remain regarding the determinant factors that affect AKI and its poor outcomes [13]. Children with AKI who progress to the stage at which renal replacement therapy would be indicated die because dialysis is simply not available or affordable in this setting [14]. Higher mortality is likely caused by lack of awareness, delayed recognition and diagnosis of AKI, late hospital presentation, and limited dialysis resources [14, 17].

Moreover, data regarding the risk of AKI in LMICs are limited. Understanding the factors associated with AKI and its early diagnosis is very important for the development of preventive and therapeutic strategies. Therefore the objective of this study was to assess the risk factors for AKI among critically ill children admitted to the PICU at the Tikur Anbessa Specialized Hospital, Addis Ababa, Ethiopia.

## Methods

### Study setting and design

This retrospective, facility-based, unmatched case-control study was conducted at Tikur Anbessa Specialized Hospital, Addis Ababa, Ethiopia. All children aged one month to 18 years admitted to the PICU at the Tikur Anbessa Specialized Hospital between January 2011 and December 2021 were included in this study.

### Population

Children with confirmed AKI cases and unmatched non-AKI children admitted to the PICU at Tikur Anbessa Specialized Hospital during the study period were included. However, we excluded children with short intensive care unit stays (< 24 h) and those who had no laboratory investigation of serum creatinine or urine output measurements during their PICU admission. Moreover, we excluded children with known chronic renal diseases and those who had undergone renal transplantation.

### Sample size and procedure

The sample size was computed using Statcalc software. The software application EPI-Info version 7.2.5.0. with the following assumptions: the proportion of mortality among controls was 8.7% and 26.5% of the cases [21], 95% confidence interval, 90% power of the study, control to case ratio of 2:1, and odds ratio of 3.78. We added a 10% non-response rate. Thus, the required sample size was 253 (85 cases and 168 controls).

The study was conducted at the Tikur Anbessa specialized hospital in Addis Ababa, Ethiopia, and the data were collected from the PICU. All the patients (cases) were critically ill children with confirmed AKI who were admitted to the PICU between January 2011 and December 2021. For each case, two controls were selected using systematic random sampling. According to data obtained from the PICU of the Tikur Anbessa specialized hospital, 2468 were admitted, of which 2374 children did not have AKI and 94 had AKI. Of these, 85 cases and 168 controls were selected.

### Data collection

Data were collected through a review of medical records using a pre-tested structured data abstraction checklist developed and validated by experts. The review checklist contained sociodemographic and morbidity mortality-related factors. It also included mechanical ventilation, radiologic contrast, and length of hospital stay. **Case**: Children with ≥ 0.3 mg/dL or ≥ 26.5 mmol/L serum creatinine level increase from the baseline within 48 h, or 1.5 to 1.9 times increment in serum creatinine from the baseline within 7 days, or urine output < 0.5 ml/kg/h for 6-12 h [2]. **Control**: Children who had no increment in serum creatinine level or urine output and did not fulfill for AKI using kidney disease improving global outcome criteria. **Baseline creatinine**: Take as normal serum creatinine of children to the age; 0.2–0.4 mg/dL for infants; 0.3–0.7 mg/dL for 1–12 years; 0.5- 1.0 mg/dL for 13–18 years [22, 23].

#### Critically ill child

Child with two or more of the following signs and symptoms such as; impaired consciousness, shock, severe respiratory distress; (severe lower chest wall in drawing, cyanosis, grunting, stridor/wheezes, hypoxemia of SPO2 < 88% or those in gasping or apneic state) severe dehydration, generalized edema, acute bleeding, severe burn, severe pallor with signs of heart failure, severe malaria, anemia < 5 g/dl, and history of two or more episodes of convulsions [24].

#### Sepsis

two or more of the following systemic inflammatory response syndrome criteria; ( fever of more than 38 °C or less than 36 °C, heart rate > 2SD per age group, respiratory > 2SD breaths per minute, abnormal white blood cell count (> 12,000/µL or < 4,000/µL), an increase in C Reactive protein (CRP), or confirmed by blood culture).

#### Nephron toxic drugs

Drugs and agents considered to cause nephrotoxicity include non-steroidal anti-inflammatory drugs, antibiotics, amphotericin-B, antiviral agents, angiotensin-converting enzyme inhibitors, calcineurin inhibitors, radiocontrast media, and cytostatic agents.

#### Hypertension

Children’s blood pressure above the 95th percentile for the same age, sex, and height.

### Data quality control

Data quality was ensured using a wise and carefully designed standardized checklist. A pretest was carried out on 12 (5%) medical charts at St. Paul’s Hospital, Millennium Medical College, before actual data collection. The content validity index was calculated by seven experts, the result was 0.93. Two days of training were provided to all data collectors and supervisors. The data collection process was closely supervised and the completeness of each questionnaire was checked daily by supervisors and the principal investigator. During data cleaning, a logical checking technique was employed to identify errors. Finally, double data entry was performed to verify the data consistency.

### Data processing and analysis

Data were entered using Epi data 3.1 version and analyzed using SPSS software 25.0 version. Cross-tabulation was performed to determine the sample characteristics. Descriptive statistics were used to describe the characteristics using tables, figures, and text. AKI was labeled as yes (coded as 1) for cases and no for controls (coded as 0). Factors associated with AKI were analyzed using a binary logistic regression model. All variables with a p-value < 0.25 in the bi-variable regression were added to the multivariable analysis after checking for multicollinearity. Associations were described using an adjusted odds ratios (aOR) and with a 95% confidence interval (CI). Multi-collinearity was checked using a variance inflation factor (> 10) and a standard error (> 2). Goodness-of-fit was checked using the Hosmer-Lemeshow test (> 0.05). Finally, statistical significance was set at a p-value < 0.05.

## Results

### Socio-demographic characteristics

A total of 241 (81 cases and 160 controls) medical charts of critically ill children were completed and reviewed for this study. The remaining records were excluded from the analysis due to incomplete data and unknown clinical measurements. Of the participants; 54 (66.7%) of the cases and 100 (62.5%) of the controls were between the age of one month and five years old, with a median age of two years. Forty-five (55.6%) cases and 75(53.1%) controls were females. Twenty-five (30.9%) cases were from the Oromia region and 45 (28.1%) of the controls were from the Addis Ababa administration. Regarding the length of hospital stay of critically ill children, Fifty-three (65.4%) of cases and 116 (72.5%) controls stayed for less than 14 days in PICU, with a median length of eight days (Table 1).

**Table 1:** Socio-demographic characteristics of critically ill children admitted to PICU at TASH, Addis Ababa, Ethiopia. (n = 241)

Variable	Case (n = 81)		Control (n = 160)	
	Freq.	%	Freq.	%
**Age**				
1 month to < 5 years	54	66.7	100	62.5
5 to 9 years	17	21.0	36	22.5
≥ 10 years	10	12.3	24	15.0
**Sex**				
Male	36	44.4	85	53.1
Female	45	55.6	75	46.9
**Region**				
Addis Ababa	23	28.4	45	28.1
Oromia	25	30.9	45	28.1
Amhara	11	13.6	22	13.8
SNNPR	6	7.4	14	8.8
Tigray	9	11.1	7	4.4
Other regions *	7	8.6	27	16.9
**Length of stay at PICU**				
Less than 14 days	53	65.4	116	72.5
15 to 29 days	14	17.3	25	15.6
30 and above days	14	17.3	19	11.9

SNNRP; Southern national-nationality People of representative, PICU; pediatric intensive care unit

### Diagnosis at admission

The most common diagnoses of cases at admission were shock 52 (63%), sepsis or infection 26 (32.1%), hypertension 25 (30.9%), and nephritic syndrome 23(28.4%. Whereas 48 (30%) of children were diagnosed with shocks and 39 (24.4%) with surgery-related diseases were among the controls group (Table 2).

**Table 2:** Common childhood cause of morbidity among critically ill children admitted to PICU at TASH, Addis Ababa, Ethiopia. (n = 241)

Variables	Case (n = 81)		Control (n = 160)	
	Freq.	%	Freq.	%
Sepsis /infection	26	32.1	18	11.2
Shock	51	63.0	48	30.0
Heart failure	11	13.6	9	5.6
Diabetes mellitus	6	7.4	7	4.4
Congenital heart disease	12	14.8	13	8.1
Meningitis	10	12.3	29	18.1
Nephrotic syndrome	17	21.0	5	3.1
Nephritic syndrome	23	28.4	15	9.4
Systemic lupus erythematous	14	17.3	6	3.8
Severe malaria	7	8.6	7	4.4
Malignancy	11	13.6	16	10.0
Pneumonia	20	24.7	29	18.1
Guillain-barre Syndrome	1	1.2	16	10.0
Hypertension	25	30.9	15	9.4
Severe acute malnutrition	14	17.3	16	10.0
Surgical-related disease*	18	22.2	39	24.4
Exposure to herbal medication	29	35.8	18	11.2
Exposure to nephrotoxic drug	18	22.2	13	8.1

* Surgery-related diseases include intestinal obstruction, tracheotomy, brain abscess, megacolon disease, and diverticulosis

### Stage of acute kidney injury and treatment outcome

Of the total cases, 72 (88.9%) had stage III AKI. Only 6 (7.4%) had a full renal recovery, and 66 (81.5%) children suffered from no renal recovery (Table 3).

**Table 3:** Stage and degree of renal recovery of AKI among critically ill children admitted to PICU at TASH, Addis Ababa, Ethiopia. (n = 81)

Variables	Freq.	%
**Stage of AKI**		
AKI stage I	5	6.2
AKI stage II	4	4.9
AKI stage III	72	88.9
**Degree of renal recovery**		
Fully recovered	6	7.4
Partial recovery	6	7.4
No renal recovery	66	81.5
Unknown renal recovery	3	3.7

AKI; acute kidney injury

### Risk factors associated with acute kidney injury

Multivariable logistic regression showed that AKI was associated with sepsis or infection, shock, hypertension, nephrotoxic drugs, mechanical ventilation, and nephritic syndrome. Critically ill children diagnosed with hypertension were five times (aOR = 5.36, 95% CI: 2.06–13.93) more likely to develop AKI than their counterparts. Children who suffered from shock were four times (aOR = 3.88, 95% CI: 1.85–8.12) more likely to develop AKI compared with their counterparts. Moreover, critically ill children exposed to nephrotoxic drugs were four times (aOR = 4.09, 95% CI: 1.45–11.59) more likely to suffer from AKI than those who didn’t have exposure to nephrotoxic drugs. Furthermore, children admitted with sepsis or infections were three times (aOR = 3.36, 95% CI: 1. 42-7.99) more likely to develop AKI than their counterparts. Children diagnosed with the nephritic syndrome were three times (aOR = 2.97, 95% CI: 1.19,-7.43) more likely to have AKI compared to their counterparts. In addition, children who received mechanical ventilation intervention were two times (aOR = 2.25, 95% CI: 1.12–4.51) more likely to develop AKI compared with those who did not receive mechanical ventilation intervention (Table 4).

**Table 4:** Risk factors associated with AKI among critically ill children admitted to PICU at TASH, Addis Ababa, Ethiopia. (n = 241)

Variable	AKI		COR (95% CI)	AOR (95% CI)	P- value
	Cases	Controls			
Sepsis or infection					
Yes	26	18	3.73(1.89 34)	**3.37 (1.42–7.99)**	**0.006**
No	55	142	1	1	
**Hypertension**					
Yes	25	15	4.32(2.12–8.78)	**5.36 (2.06–13.93)**	**0.001**
No	56	145	1	1	
**Heart failure**					
Yes	11	9	2.637(1.05–6.65)	1.69 (0.44–6.44)	0.443
No	70	151	1	1	
**Hypovolemic Shock**					
Yes	51	48	3.967(2.26–6.97)	**3.876(1.85–8.12)**	**0.000**
No	30	112	1	1	
*Exposure to **Nephrotoxic drugs**					
Yes	18	13	3.23 (1.49–6.99)	**4.09 (1.45–11.59)**	**0.008**
No	63	147	1.00	1	
**Exposure to** **Herbal medication**					
Yes	29	18	4.40 (2.25–8.58)	2.38 (0.95–5.92)	0.063
No	52	142	1	1	
**Mechanical ventilation**					
Yes	52	64	2.69 (1.55–4.68)	**2.25 (1.12–4.51)**	**0.023**
No	29	96	1	1	
**Systemic lupus** **Erythematous**					
Yes	14	6	5.36 (1.97–14.56)	3.26 (0.80–13.32)	0.100
No	67	154	1	1	
**Electrolyte Abnormality**					
Yes	16	16	2.22(1.044 4.70)	1.66 (0.60–4.59)	0.325
No	65	144	1	1	
**Nephrotic- syndrome**					
Yes	14	9	3.50 (1.45–8.50)	1.56 (0.48–5.06)	0.460
No	67	151	1	1	
**Nephritic syndrome**					
Yes	23	15	3.83 (1.87–7.86)	**2.97 (1.19–7.43)**	**0.020**
No	58	145	1	1	

AKI; Acute Kidney Injury; AOR, Adjusted Odds Ratio; COR, Crude Odds Ratio; CI, Confidence interval; *exposure to nephrotoxic drugs (aminoglycoside, vancomycin, nonsteroidal anti-inflammatory drug, radiologic contrast)

## Discussion

AKI is a serious public health problem, especially in children, and a common cause of morbidity and mortality [11, 25, 26]. There is evidence that AKI is often underdiagnosed; delays in recognition and treatment can lead to death before diagnosis [27]. This study was conducted to assess the risk factors associated with AKI among critically ill children admitted to the PICU at the Tikur Anbessa Specialized Hospital in Addis Ababa, Ethiopia. We found the risk of AKI was higher among critically ill children admitted with hypertension, hypovolemic shock, nephrotoxic drug exposure, sepsis or infection, nephritic syndrome, and mechanical ventilation.

In this study, critically ill children diagnosed with hypertension were five times more likely to develop AKI than their counterparts. This is consistent with the results of a study conducted in Montreal, Canada [28]. This could be related to high blood pressure, which causes narrowing, weakening, or hardening of arteries around the kidney. Moreover, damaged arteries cannot deliver sufficient blood to the kidney tissue which resulting in a decline in renal function [29, 30].

Similarly, critically ill children admitted with hypovolemic shock were four times more likely to develop AKI than critically ill children admitted without hypovolemic shock. This study was supported by previous studies conducted in China and other countries [31–33]. This is because shock causes circulatory compromise; consequently, kidney injury develops because of ineffective renal blood flow and renal vascular ischemia, and kidney function declines as urine flow decreases and execration is completely suppressed.

Moreover, the present findings indicate that critically ill children diagnosed with sepsis or infection are three times more likely to develop AKI than their counterparts. This finding is in agreement with those of studies in China [31], Addis Ababa, Ethiopia [21], Pennsylvania, the USA, and Taiwan [34, 35]. Sepsis causes profound alteration of circulation and is characterized by impaired distribution of blood flow, decreased peripheral vascular resistance, and derangement of microcirculatory perfusions, such as the renal vasculature [36, 37]. Moreover, some evidence suggests that the inflammatory response that occurs during sepsis causes an adaptive response of the tubular epithelial cell, which is to ensure cell survival by decreasing the cellular function to reduce energy use. Therefore, the occurrence of renal inflammation and abnormal microvascular function, exacerbates the adaptive response of tubular epithelial cells to injury, and consequently declines renal function [36, 38].

Furthermore, critically ill children who had nephritic syndrome were three times more likely to develop AKI than those who didn’t have nephritic syndrome. This study is in line with the result of a previous study conducted in China [32]. Nephritic syndrome is an immunological complication of infection with group A β-hemolytic streptococcus, the immunological complex where deposited in the membranous of glomeruli it results in damaging the underlying tissue and impaired glomerular filtration, it is characterized by microscopic or gross hematuria, hypertension, edema, oliguria, and AKI occurs as a complication of post-streptococcal glomerular nephritis [39].

In addition, the current findings showed that exposure to nephrotoxic drugs such as aminoglycosides, vancomycin, non-steroidal anti-inflammatory drugs, and radiologic contrast were four times greater risk to develop AKI than their counterparts. This finding was supported by studies conducted in India, Toronto Canada, Ethiopia, and Italy [28, 40, 41]. This is because of the decline in detoxification and excretion of kidney function as a result of kidney injury caused by endogenous or exogenous toxicants [42]. Another justification indicated that the exposure of nephrons to drugs often results in alteration of the regulatory mechanism, including impaired glomerular filtration rate, and induces inflammation surrounding the glomerulus, proximal tubules, and cellular matrix [43].

According to the present findings, critically ill children who received mechanical ventilation intervention were twice as likely to develop AKI compared to those who did not receive mechanical ventilation. This finding is in line with studies conducted in Canada and Taiwan [11, 35]. Mechanical ventilation-induced AKI resulting from renal tissue perfusion is impaired due to changes in hemodynamics [44]. Another explanation is that the chemical is released from neurohumoral-mediated changes in intrarenal blood flow [42]. In addition, some studies have shown that systemic inflammatory mediators produced by ventilator-induced lung injury damage kidney tissue through systemic circulation [45]. Moreover, critically ill children who are on mechanical ventilation may experience prolonged hospital stays, which is indeed associated with AKI [46].

We believe that the evidence generated from this study has implications for local policymakers, researchers, and clinicians to give special attention to reducing preventable AKI-associated deaths through the betterment of infrastructure and more prospective research is needed in LMICs for the prevention and early treatment of AKI to save more lives.

### Limitations

Our study has some limitations. First, the findings are based on a retrospective review of medical records; therefore, some important variables may be missed. Second, this is an institutional study focusing on patients with AKI admitted to the PICU and may not be generalizable to other populations. Third, the availability of baseline creatinine data was challenging. We calculated the baseline based on the assumption of normal renal function which has been used in different studies.

## Conclusion

In conclusion, the diagnosis of sepsis or infection, hypertension, shock, exposure to nephrotoxic drugs, demand for mechanical ventilation support, and nephritic syndrome were factors that increased the risk of AKI in critically ill children. Multiple risk factors for AKI are associated with illness and its severity. All measures that ensure adequate renal perfusion must be taken in children with identified risk factors to prevent the development of AKI. Further studies with larger sample sizes and high-level study designs are recommended to better understand the risk factors.

## Data Availability

All data of this study are available from the corresponding author upon reasonable request.

## List of abbreviations

AKI: Acute Kidney Injury
AOR: adjusted Odd Ratio
AWARE: Assessment of Worldwide Acute Kidney Injury Renal Angina Epidemiology
COR: Crude Odd Ratio
CRP: C-reactive protein
KDIGO: Kidney Disease Improving Global Outcome
LMICs: Low-middle-income countries
PICU: Pediatric Intensive Care Unit
SPSS: Statistical Package for Social Sciences
TASH: Tikur Anbessa Specialized Hospital
